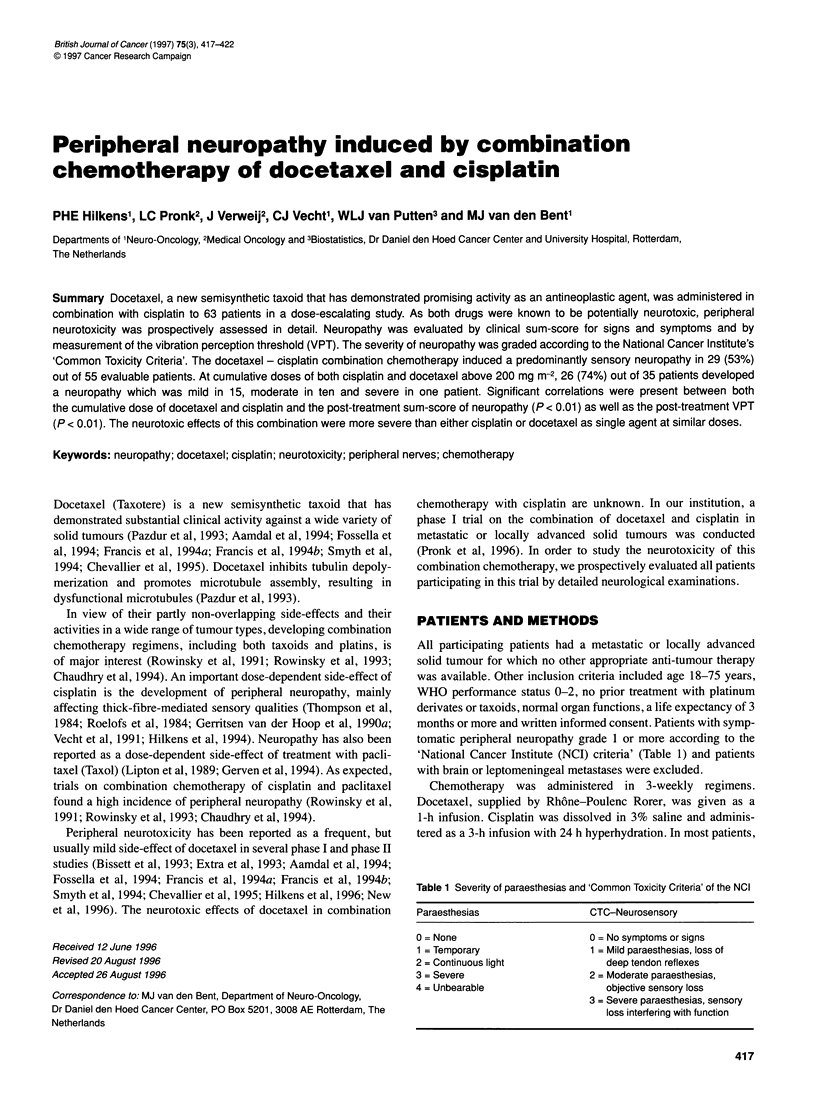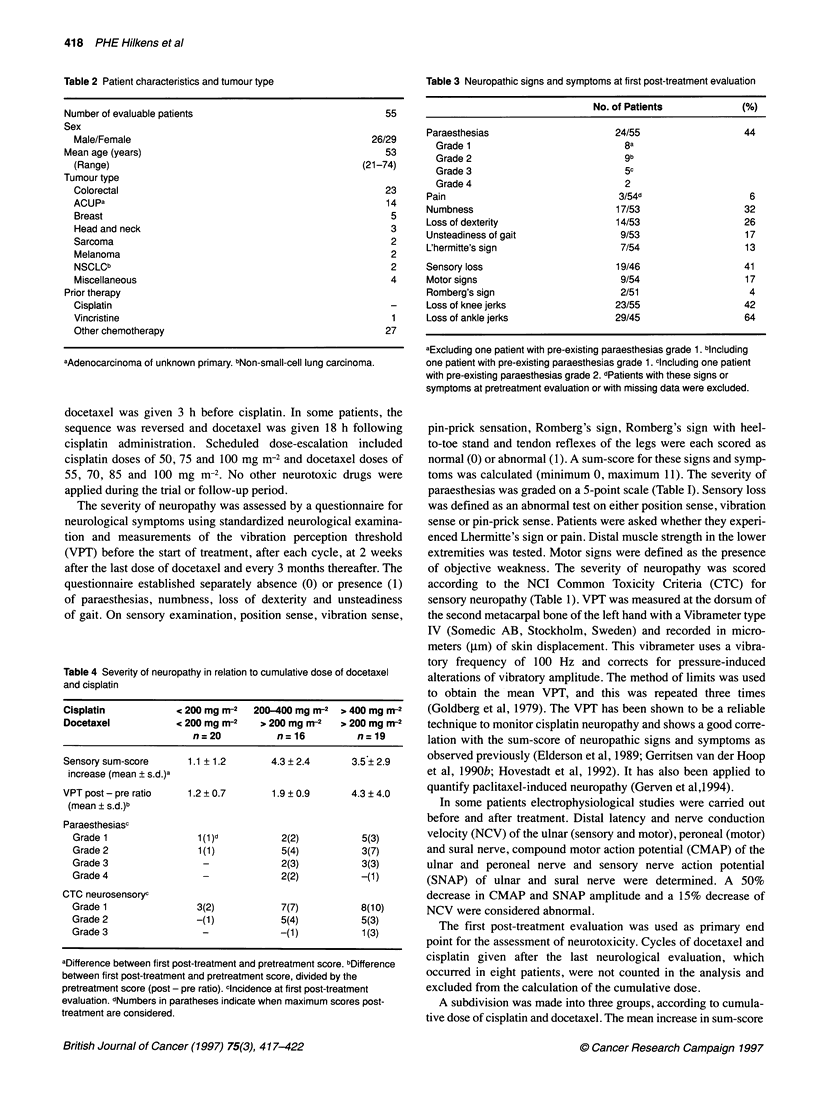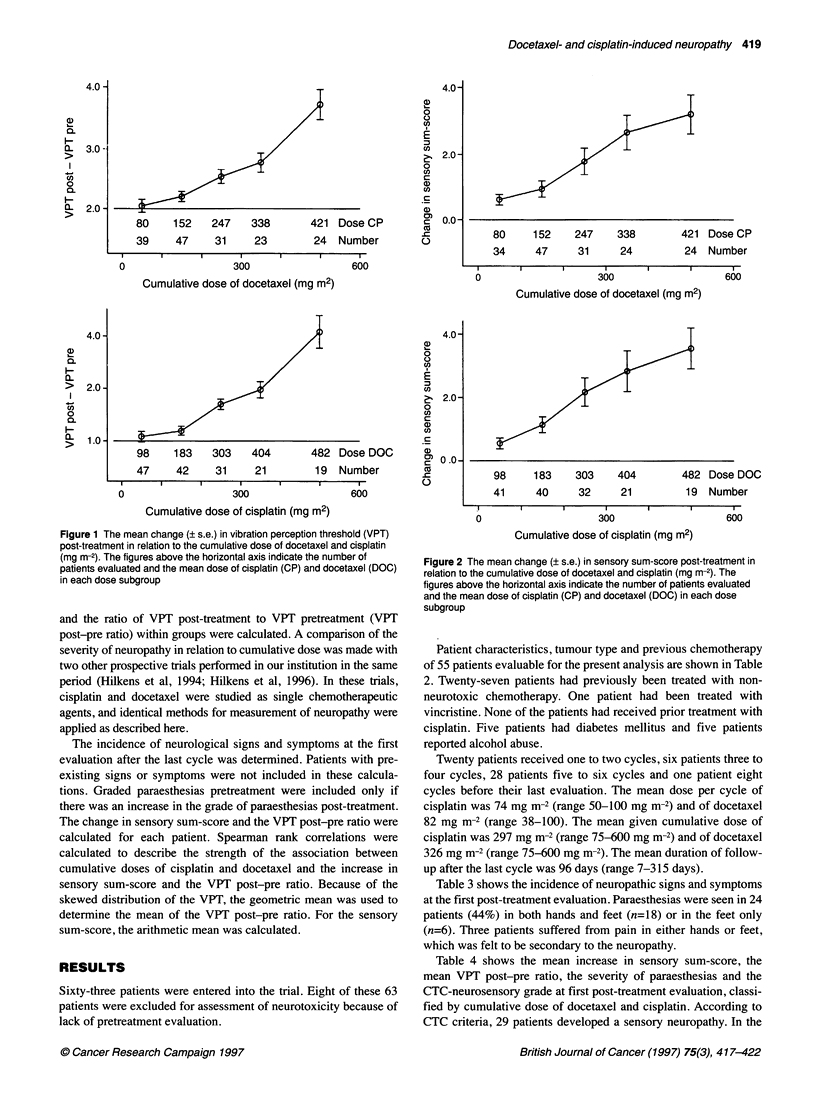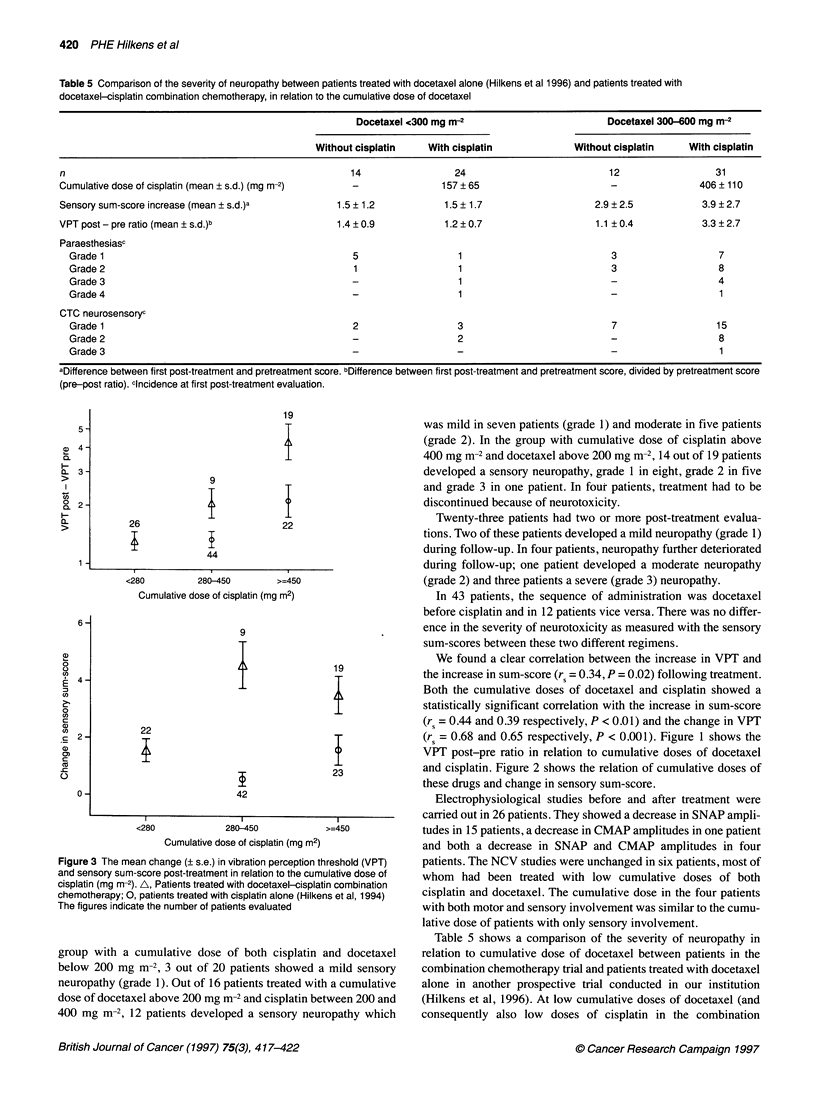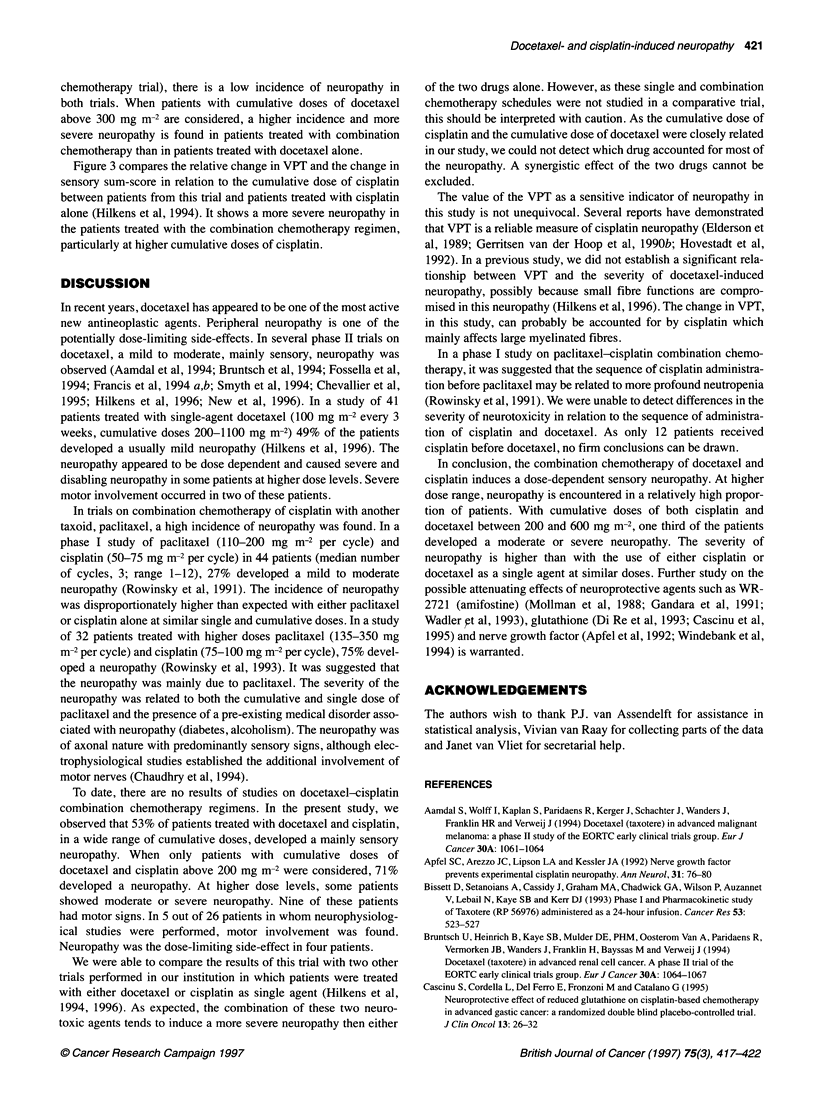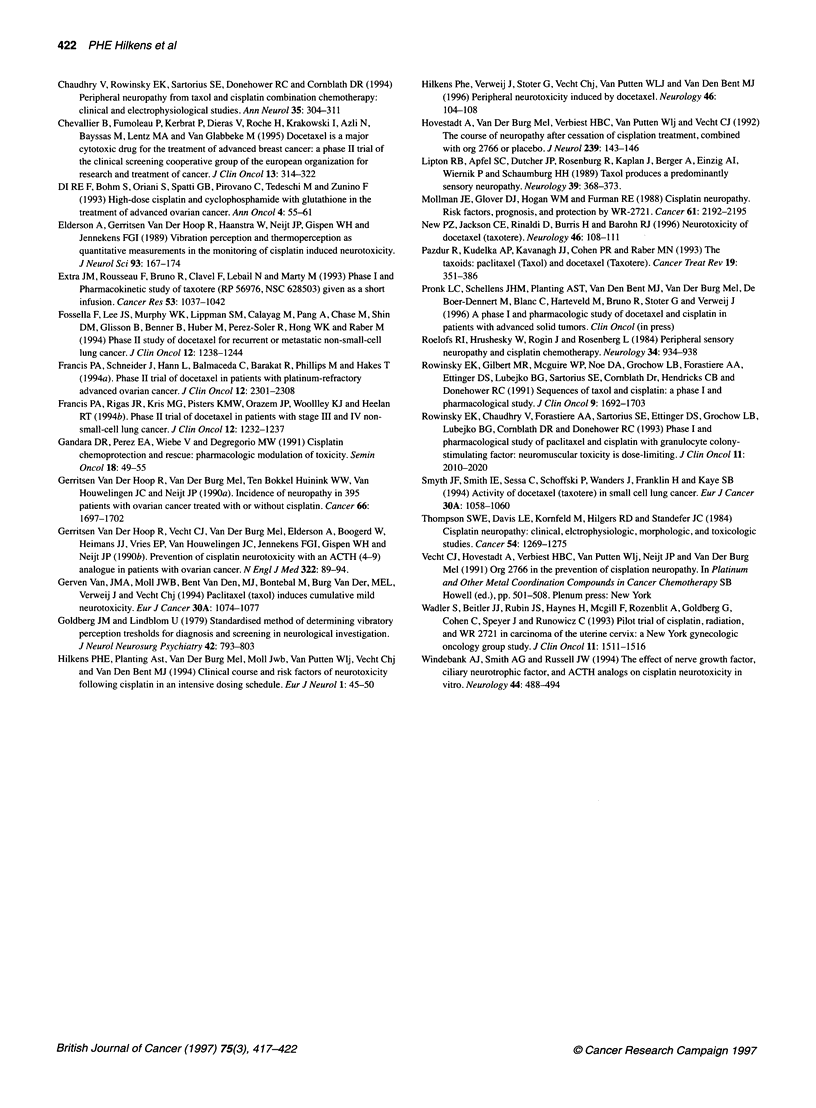# Peripheral neuropathy induced by combination chemotherapy of docetaxel and cisplatin.

**DOI:** 10.1038/bjc.1997.68

**Published:** 1997

**Authors:** P. H. Hilkens, L. C. Pronk, J. Verweij, C. J. Vecht, W. L. van Putten, M. J. van den Bent

**Affiliations:** Department of Neuro-Oncology, Dr Daniel den Hoed Cancer Center and University Hospital, Rotterdam, The Netherlands.

## Abstract

Docetaxel, a new semisynthetic taxoid that has demonstrated promising activity as an antineoplastic agent, was administered in combination with cisplatin to 63 patients in a dose-escalating study. As both drugs were known to be potentially neurotoxic, peripheral neurotoxicity was prospectively assessed in detail. Neuropathy was evaluated by clinical sum-score for signs and symptoms and by measurement of the vibration perception threshold (VPT). The severity of neuropathy was graded according to the National Cancer Institute's 'Common Toxicity Criteria'. The docetaxel-cisplatin combination chemotherapy induced a predominantly sensory neuropathy in 29 (53%) out of 55 evaluable patients. At cumulative doses of both cisplatin and docetaxel above 200 mg m(-2), 26 (74%) out of 35 patients developed a neuropathy which was mild in 15, moderate in ten and severe in one patient. Significant correlations were present between both the cumulative dose of docetaxel and cisplatin and the post-treatment sum-score of neuropathy (P < 0.01) as well as the post-treatment VPT (P < 0.01). The neurotoxic effects of this combination were more severe than either cisplatin or docetaxel as single agent at similar doses.


*British Journal of Cancer* (1997) **75**(3), 417–422
© 1997 Cancer Research Campaign

### Peripheral neuropathy induced by combination chemotherapy of docetaxel and cisplatin

**PHE Hilkens[1], LC Pronk[2], J Verweij[2], CJ Vecht[1], WLJ van Putten[3] and MJ van den Bent[1]**

Departments of [1]Neuro-Oncology, [2]Medical Oncology and [3]Biostatistics, Dr Daniel den Hoed Cancer Center and University Hospital, Rotterdam, The Netherlands

**Summary** Docetaxel, a new semisynthetic taxoid that has demonstrated promising activity as an antineoplastic agent, was administered in combination with cisplatin to 63 patients in a dose-escalating study. As both drugs were known to be potentially neurotoxic, peripheral neurotoxicity was prospectively assessed in detail. Neuropathy was evaluated by clinical sum-score for signs and symptoms and by measurement of the vibration perception threshold (VPT). The severity of neuropathy was graded according to the National Cancer Institute's 'Common Toxicity Criteria'. The docetaxel – cisplatin combination chemotherapy induced a predominantly sensory neuropathy in 29 (53%) out of 55 evaluable patients. At cumulative doses of both cisplatin and docetaxel above 200 mg $m^{-2}$, 26 (74%) out of 35 patients developed a neuropathy which was mild in 15, moderate in ten and severe in one patient. Significant correlations were present between both the cumulative dose of docetaxel and cisplatin and the post-treatment sum-score of neuropathy ($P < 0.01$) as well as the post-treatment VPT ($P < 0.01$). The neurotoxic effects of this combination were more severe than either cisplatin or docetaxel as single agent at similar doses.

**Keywords:** neuropathy; docetaxel; cisplatin; neurotoxicity; peripheral nerves; chemotherapy

Docetaxel (Taxotere) is a new semisynthetic taxoid that has demonstrated substantial clinical activity against a wide variety of solid tumours (Pazdur et al, 1993; Aamdal et al, 1994; Fossella et al, 1994; Francis et al, 1994*a*; Francis et al, 1994*b*; Smyth et al, 1994; Chevallier et al, 1995). Docetaxel inhibits tubulin depolymerization and promotes microtubule assembly, resulting in dysfunctional microtubules (Pazdur et al, 1993).

In view of their partly non-overlapping side-effects and their activities in a wide range of tumour types, developing combination chemotherapy regimens, including both taxoids and platins, is of major interest (Rowinsky et al, 1991; Rowinsky et al, 1993; Chaudhry et al, 1994). An important dose-dependent side-effect of cisplatin is the development of peripheral neuropathy, mainly affecting thick-fibre-mediated sensory qualities (Thompson et al, 1984; Roelofs et al, 1984; Gerritsen van der Hoop et al, 1990*a*; Vecht et al, 1991; Hilkens et al, 1994). Neuropathy has also been reported as a dose-dependent side-effect of treatment with paclitaxel (Taxol) (Lipton et al, 1989; Gerven et al, 1994). As expected, trials on combination chemotherapy of cisplatin and paclitaxel found a high incidence of peripheral neuropathy (Rowinsky et al, 1991; Rowinsky et al, 1993; Chaudhry et al, 1994).

Peripheral neurotoxicity has been reported as a frequent, but usually mild side-effect of docetaxel in several phase I and phase II studies (Bissett et al, 1993; Extra et al, 1993; Aamdal et al, 1994; Fossella et al, 1994; Francis et al, 1994*a*; Francis et al, 1994*b*; Smyth et al, 1994; Chevallier et al, 1995; Hilkens et al, 1996; New et al, 1996). The neurotoxic effects of docetaxel in combination chemotherapy with cisplatin are unknown. In our institution, a phase I trial on the combination of docetaxel and cisplatin in metastatic or locally advanced solid tumours was conducted (Pronk et al, 1996). In order to study the neurotoxicity of this combination chemotherapy, we prospectively evaluated all patients participating in this trial by detailed neurological examinations.

*Received 12 June 1996*
*Revised 20 August 1996*
*Accepted 26 August 1996*

*Correspondence to:* MJ van den Bent, Department of Neuro-Oncology, Dr Daniel den Hoed Cancer Center, PO Box 5201, 3008 AE Rotterdam, The Netherlands

### PATIENTS AND METHODS

All participating patients had a metastatic or locally advanced solid tumour for which no other appropriate anti-tumour therapy was available. Other inclusion criteria included age 18–75 years, WHO performance status 0–2, no prior treatment with platinum derivates or taxoids, normal organ functions, a life expectancy of 3 months or more and written informed consent. Patients with symptomatic peripheral neuropathy grade 1 or more according to the 'National Cancer Institute (NCI) criteria' (Table 1) and patients with brain or leptomeningeal metastases were excluded.

Chemotherapy was administered in 3-weekly regimens. Docetaxel, supplied by Rhône–Poulenc Rorer, was given as a 1-h infusion. Cisplatin was dissolved in 3% saline and administered as a 3-h infusion with 24 h hyperhydration. In most patients,

**Table 1** Severity of paraesthesias and 'Common Toxicity Criteria' of the NCI

| Paraesthesias | CTC–Neurosensory |
|---|---|
| 0 = None | 0 = No symptoms or signs |
| 1 = Temporary | 1 = Mild paraesthesias, loss of deep tendon reflexes |
| 2 = Continuous light | 2 = Moderate paraesthesias, objective sensory loss |
| 3 = Severe | 3 = Severe paraesthesias, sensory loss interfering with function |
| 4 = Unbearable | |

Table 2 Patient characteristics and tumour type

| | |
|---|---|
| Number of evaluable patients | 55 |
| Sex | |
| Male/Female | 26/29 |
| Mean age (years) | 53 |
| (Range) | (21–74) |
| Tumour type | |
| Colorectal | 23 |
| ACUP[a] | 14 |
| Breast | 5 |
| Head and neck | 3 |
| Sarcoma | 2 |
| Melanoma | 2 |
| NSCLC[b] | 2 |
| Miscellaneous | 4 |
| Prior therapy | |
| Cisplatin | – |
| Vincristine | 1 |
| Other chemotherapy | 27 |

[a]Adenocarcinoma of unknown primary. [b]Non-small-cell lung carcinoma.

Table 3 Neuropathic signs and symptoms at first post-treatment evaluation

| | No. of Patients | (%) |
|---|---|---|
| Paraesthesias | 24/55 | 44 |
| Grade 1 | 8[a] | |
| Grade 2 | 9[b] | |
| Grade 3 | 5[c] | |
| Grade 4 | 2 | |
| Pain | 3/54[d] | 6 |
| Numbness | 17/53 | 32 |
| Loss of dexterity | 14/53 | 26 |
| Unsteadiness of gait | 9/53 | 17 |
| L'hermitte's sign | 7/54 | 13 |
| Sensory loss | 19/46 | 41 |
| Motor signs | 9/54 | 17 |
| Romberg's sign | 2/51 | 4 |
| Loss of knee jerks | 23/55 | 42 |
| Loss of ankle jerks | 29/45 | 64 |

[a]Excluding one patient with pre-existing paraesthesias grade 1. [b]Including one patient with pre-existing paraesthesias grade 1. [c]Including one patient with pre-existing paraesthesias grade 2. [d]Patients with these signs or symptoms at pretreatment evaluation or with missing data were excluded.

docetaxel was given 3 h before cisplatin. In some patients, the sequence was reversed and docetaxel was given 18 h following cisplatin administration. Scheduled dose-escalation included cisplatin doses of 50, 75 and 100 mg $m^{-2}$ and docetaxel doses of 55, 70, 85 and 100 mg $m^{-2}$. No other neurotoxic drugs were applied during the trial or follow-up period.

The severity of neuropathy was assessed by a questionnaire for neurological symptoms using standardized neurological examination and measurements of the vibration perception threshold (VPT) before the start of treatment, after each cycle, at 2 weeks after the last dose of docetaxel and every 3 months thereafter. The questionnaire established separately absence (0) or presence (1) of paraesthesias, numbness, loss of dexterity and unsteadiness of gait. On sensory examination, position sense, vibration sense, pin-prick sensation, Romberg's sign, Romberg's sign with heel-to-toe stand and tendon reflexes of the legs were each scored as normal (0) or abnormal (1). A sum-score for these signs and symptoms was calculated (minimum 0, maximum 11). The severity of paraesthesias was graded on a 5-point scale (Table I). Sensory loss was defined as an abnormal test on either position sense, vibration sense or pin-prick sense. Patients were asked whether they experienced Lhermitte's sign or pain. Distal muscle strength in the lower extremities was tested. Motor signs were defined as the presence of objective weakness. The severity of neuropathy was scored according to the NCI Common Toxicity Criteria (CTC) for sensory neuropathy (Table 1). VPT was measured at the dorsum of the second metacarpal bone of the left hand with a Vibrameter type IV (Somedic AB, Stockholm, Sweden) and recorded in micrometers (μm) of skin displacement. This vibrameter uses a vibratory frequency of 100 Hz and corrects for pressure-induced alterations of vibratory amplitude. The method of limits was used to obtain the mean VPT, and this was repeated three times (Goldberg et al, 1979). The VPT has been shown to be a reliable technique to monitor cisplatin neuropathy and shows a good correlation with the sum-score of neuropathic signs and symptoms as observed previously (Elderson et al, 1989; Gerritsen van der Hoop et al, 1990*b*; Hovestadt et al, 1992). It has also been applied to quantify paclitaxel-induced neuropathy (Gerven et al,1994).

Table 4 Severity of neuropathy in relation to cumulative dose of docetaxel and cisplatin

| **Cisplatin** <br> **Docetaxel** | **< 200 mg m⁻²** <br> **< 200 mg m⁻²** <br> ***n* = 20** | **200–400 mg m⁻²** <br> **> 200 mg m⁻²** <br> ***n* = 16** | **> 400 mg m⁻²** <br> **> 200 mg m⁻²** <br> ***n* = 19** |
|---|---|---|---|
| Sensory sum-score increase (mean ± s.d.)[a] | 1.1 ± 1.2 | 4.3 ± 2.4 | 3.5 ± 2.9 |
| VPT post – pre ratio (mean ± s.d.)[b] | 1.2 ± 0.7 | 1.9 ± 0.9 | 4.3 ± 4.0 |
| Paraesthesias[c] | | | |
| Grade 1 | 1(1)[d] | 2(2) | 5(3) |
| Grade 2 | 1(1) | 5(4) | 3(7) |
| Grade 3 | – | 2(3) | 3(3) |
| Grade 4 | – | 2(2) | –(1) |
| CTC neurosensory[c] | | | |
| Grade 1 | 3(2) | 7(7) | 8(10) |
| Grade 2 | –(1) | 5(4) | 5(3) |
| Grade 3 | – | –(1) | 1(3) |

[a]Difference between first post-treatment and pretreatment score. [b]Difference between first post-treatment and pretreatment score, divided by the pretreatment score (post – pre ratio). [c]Incidence at first post-treatment evaluation. [d]Numbers in paratheses indicate when maximum scores post-treatment are considered.

In some patients electrophysiological studies were carried out before and after treatment. Distal latency and nerve conduction velocity (NCV) of the ulnar (sensory and motor), peroneal (motor) and sural nerve, compound motor action potential (CMAP) of the ulnar and peroneal nerve and sensory nerve action potential (SNAP) of ulnar and sural nerve were determined. A 50% decrease in CMAP and SNAP amplitude and a 15% decrease of NCV were considered abnormal.

The first post-treatment evaluation was used as primary end point for the assessment of neurotoxicity. Cycles of docetaxel and cisplatin given after the last neurological evaluation, which occurred in eight patients, were not counted in the analysis and excluded from the calculation of the cumulative dose.

A subdivision was made into three groups, according to cumulative dose of cisplatin and docetaxel. The mean increase in sum-score

© Cancer Research Campaign 1997

**Figure 1** The mean change (± s.e.) in vibration perception threshold (VPT) post-treatment in relation to the cumulative dose of docetaxel and cisplatin (mg m$^{-2}$). The figures above the horizontal axis indicate the number of patients evaluated and the mean dose of cisplatin (CP) and docetaxel (DOC) in each dose subgroup

**Figure 2** The mean change (± s.e.) in sensory sum-score post-treatment in relation to the cumulative dose of docetaxel and cisplatin (mg m$^{-2}$). The figures above the horizontal axis indicate the number of patients evaluated and the mean dose of cisplatin (CP) and docetaxel (DOC) in each dose subgroup and the ratio of VPT post-treatment to VPT pretreatment (VPT post–pre ratio) within groups were calculated. A comparison of the severity of neuropathy in relation to cumulative dose was made with two other prospective trials performed in our institution in the same period (Hilkens et al, 1994; Hilkens et al, 1996). In these trials, cisplatin and docetaxel were studied as single chemotherapeutic agents, and identical methods for measurement of neuropathy were applied as described here.

The incidence of neurological signs and symptoms at the first evaluation after the last cycle was determined. Patients with pre-existing signs or symptoms were not included in these calculations. Graded paraesthesias pretreatment were included only if there was an increase in the grade of paraesthesias post-treatment. The change in sensory sum-score and the VPT post–pre ratio were calculated for each patient. Spearman rank correlations were calculated to describe the strength of the association between cumulative doses of cisplatin and docetaxel and the increase in sensory sum-score and the VPT post–pre ratio. Because of the skewed distribution of the VPT, the geometric mean was used to determine the mean of the VPT post–pre ratio. For the sensory sum-score, the arithmetic mean was calculated.

### RESULTS

Sixty-three patients were entered into the trial. Eight of these 63 patients were excluded for assessment of neurotoxicity because of lack of pretreatment evaluation.

Patient characteristics, tumour type and previous chemotherapy of 55 patients evaluable for the present analysis are shown in Table 2. Twenty-seven patients had previously been treated with non-neurotoxic chemotherapy. One patient had been treated with vincristine. None of the patients had received prior treatment with cisplatin. Five patients had diabetes mellitus and five patients reported alcohol abuse.

Twenty patients received one to two cycles, six patients three to four cycles, 28 patients five to six cycles and one patient eight cycles before their last evaluation. The mean dose per cycle of cisplatin was 74 mg m$^{-2}$ (range 50–100 mg m$^{-2}$) and of docetaxel 82 mg m$^{-2}$ (range 38–100). The mean given cumulative dose of cisplatin was 297 mg m$^{-2}$ (range 75–600 mg m$^{-2}$) and of docetaxel 326 mg m$^{-2}$ (range 75–600 mg m$^{-2}$). The mean duration of follow-up after the last cycle was 96 days (range 7–315 days).

Table 3 shows the incidence of neuropathic signs and symptoms at the first post-treatment evaluation. Paraesthesias were seen in 24 patients (44%) in both hands and feet ($n$=18) or in the feet only ($n$=6). Three patients suffered from pain in either hands or feet, which was felt to be secondary to the neuropathy.

Table 4 shows the mean increase in sensory sum-score, the mean VPT post–pre ratio, the severity of paraesthesias and the CTC-neurosensory grade at first post-treatment evaluation, classified by cumulative dose of docetaxel and cisplatin. According to CTC criteria, 29 patients developed a sensory neuropathy. In the

© Cancer Research Campaign 1997

**Table 5** Comparison of the severity of neuropathy between patients treated with docetaxel alone (Hilkens et al 1996) and patients treated with docetaxel–cisplatin combination chemotherapy, in relation to the cumulative dose of docetaxel

| | Docetaxel <300 mg m^-2 | | Docetaxel 300–600 mg m^-2 | |
|---|---|---|---|---|
| | **Without cisplatin** | **With cisplatin** | **Without cisplatin** | **With cisplatin** |
| *n* | 14 | 24 | 12 | 31 |
| Cumulative dose of cisplatin (mean ± s.d.) (mg m^-2) | – | 157 ± 65 | – | 406 ± 110 |
| Sensory sum-score increase (mean ± s.d.)[a] | 1.5 ± 1.2 | 1.5 ± 1.7 | 2.9 ± 2.5 | 3.9 ± 2.7 |
| VPT post – pre ratio (mean ± s.d.)[b] | 1.4 ± 0.9 | 1.2 ± 0.7 | 1.1 ± 0.4 | 3.3 ± 2.7 |
| Paraesthesias[c] | | | | |
| Grade 1 | 5 | 1 | 3 | 7 |
| Grade 2 | 1 | 1 | 3 | 8 |
| Grade 3 | – | 1 | – | 4 |
| Grade 4 | – | 1 | – | 1 |
| CTC neurosensory[c] | | | | |
| Grade 1 | 2 | 3 | 7 | 15 |
| Grade 2 | – | 2 | – | 8 |
| Grade 3 | – | – | – | 1 |

[a]Difference between first post-treatment and pretreatment score. [b]Difference between first post-treatment and pretreatment score, divided by pretreatment score (pre–post ratio). [c]Incidence at first post-treatment evaluation.

**Figure 3** The mean change (± s.e.) in vibration perception threshold (VPT) and sensory sum-score post-treatment in relation to the cumulative dose of cisplatin (mg m$^{-2}$). △, Patients treated with docetaxel–cisplatin combination chemotherapy; O, patients treated with cisplatin alone (Hilkens et al, 1994) The figures indicate the number of patients evaluated group with a cumulative dose of both cisplatin and docetaxel below 200 mg m$^{-2}$, 3 out of 20 patients showed a mild sensory neuropathy (grade 1). Out of 16 patients treated with a cumulative dose of docetaxel above 200 mg m$^{-2}$ and cisplatin between 200 and 400 mg m$^{-2}$, 12 patients developed a sensory neuropathy which was mild in seven patients (grade 1) and moderate in five patients (grade 2). In the group with cumulative dose of cisplatin above 400 mg m$^{-2}$ and docetaxel above 200 mg m$^{-2}$, 14 out of 19 patients developed a sensory neuropathy, grade 1 in eight, grade 2 in five and grade 3 in one patient. In four patients, treatment had to be discontinued because of neurotoxicity.

Twenty-three patients had two or more post-treatment evaluations. Two of these patients developed a mild neuropathy (grade 1) during follow-up. In four patients, neuropathy further deteriorated during follow-up; one patient developed a moderate neuropathy (grade 2) and three patients a severe (grade 3) neuropathy.

In 43 patients, the sequence of administration was docetaxel before cisplatin and in 12 patients vice versa. There was no difference in the severity of neurotoxicity as measured with the sensory sum-scores between these two different regimens.

We found a clear correlation between the increase in VPT and the increase in sum-score ($r_s = 0.34, P = 0.02$) following treatment. Both the cumulative doses of docetaxel and cisplatin showed a statistically significant correlation with the increase in sum-score ($r_s$ = 0.44 and 0.39 respectively, $P < 0.01$) and the change in VPT ($r_s$ = 0.68 and 0.65 respectively, $P < 0.001$). Figure 1 shows the VPT post–pre ratio in relation to cumulative doses of docetaxel and cisplatin. Figure 2 shows the relation of cumulative doses of these drugs and change in sensory sum-score.

Electrophysiological studies before and after treatment were carried out in 26 patients. They showed a decrease in SNAP amplitudes in 15 patients, a decrease in CMAP amplitudes in one patient and both a decrease in SNAP and CMAP amplitudes in four patients. The NCV studies were unchanged in six patients, most of whom had been treated with low cumulative doses of both cisplatin and docetaxel. The cumulative dose in the four patients with both motor and sensory involvement was similar to the cumulative dose of patients with only sensory involvement.

Table 5 shows a comparison of the severity of neuropathy in relation to cumulative dose of docetaxel between patients in the combination chemotherapy trial and patients treated with docetaxel alone in another prospective trial conducted in our institution (Hilkens et al, 1996). At low cumulative doses of docetaxel (and consequently also low doses of cisplatin in the combination

© Cancer Research Campaign 1997

chemotherapy trial), there is a low incidence of neuropathy in both trials. When patients with cumulative doses of docetaxel above 300 mg $m^{-2}$ are considered, a higher incidence and more severe neuropathy is found in patients treated with combination chemotherapy than in patients treated with docetaxel alone.

Figure 3 compares the relative change in VPT and the change in sensory sum-score in relation to the cumulative dose of cisplatin between patients from this trial and patients treated with cisplatin alone (Hilkens et al, 1994). It shows a more severe neuropathy in the patients treated with the combination chemotherapy regimen, particularly at higher cumulative doses of cisplatin.

### DISCUSSION

In recent years, docetaxel has appeared to be one of the most active new antineoplastic agents. Peripheral neuropathy is one of the potentially dose-limiting side-effects. In several phase II trials on docetaxel, a mild to moderate, mainly sensory, neuropathy was observed (Aamdal et al, 1994; Bruntsch et al, 1994; Fossella et al, 1994; Francis et al, 1994 *a,b*; Smyth et al, 1994; Chevallier et al, 1995; Hilkens et al, 1996; New et al, 1996). In a study of 41 patients treated with single-agent docetaxel (100 mg $m^{-2}$ every 3 weeks, cumulative doses 200–1100 mg $m^{-2}$) 49% of the patients developed a usually mild neuropathy (Hilkens et al, 1996). The neuropathy appeared to be dose dependent and caused severe and disabling neuropathy in some patients at higher dose levels. Severe motor involvement occurred in two of these patients.

In trials on combination chemotherapy of cisplatin with another taxoid, paclitaxel, a high incidence of neuropathy was found. In a phase I study of paclitaxel (110–200 mg $m^{-2}$ per cycle) and cisplatin (50–75 mg $m^{-2}$ per cycle) in 44 patients (median number of cycles, 3; range 1–12), 27% developed a mild to moderate neuropathy (Rowinsky et al, 1991). The incidence of neuropathy was disproportionately higher than expected with either paclitaxel or cisplatin alone at similar single and cumulative doses. In a study of 32 patients treated with higher doses paclitaxel (135–350 mg $m^{-2}$ per cycle) and cisplatin (75–100 mg $m^{-2}$ per cycle), 75% developed a neuropathy (Rowinsky et al, 1993). It was suggested that the neuropathy was mainly due to paclitaxel. The severity of the neuropathy was related to both the cumulative and single dose of paclitaxel and the presence of a pre-existing medical disorder associated with neuropathy (diabetes, alcoholism). The neuropathy was of axonal nature with predominantly sensory signs, although electrophysiological studies established the additional involvement of motor nerves (Chaudhry et al, 1994).

To date, there are no results of studies on docetaxel–cisplatin combination chemotherapy regimens. In the present study, we observed that 53% of patients treated with docetaxel and cisplatin, in a wide range of cumulative doses, developed a mainly sensory neuropathy. When only patients with cumulative doses of docetaxel and cisplatin above 200 mg $m^{-2}$ were considered, 71% developed a neuropathy. At higher dose levels, some patients showed moderate or severe neuropathy. Nine of these patients had motor signs. In 5 out of 26 patients in whom neurophysiological studies were performed, motor involvement was found. Neuropathy was the dose-limiting side-effect in four patients.

We were able to compare the results of this trial with two other trials performed in our institution in which patients were treated with either docetaxel or cisplatin as single agent (Hilkens et al, 1994, 1996). As expected, the combination of these two neurotoxic agents tends to induce a more severe neuropathy then either of the two drugs alone. However, as these single and combination chemotherapy schedules were not studied in a comparative trial, this should be interpreted with caution. As the cumulative dose of cisplatin and the cumulative dose of docetaxel were closely related in our study, we could not detect which drug accounted for most of the neuropathy. A synergistic effect of the two drugs cannot be excluded.

The value of the VPT as a sensitive indicator of neuropathy in this study is not unequivocal. Several reports have demonstrated that VPT is a reliable measure of cisplatin neuropathy (Elderson et al, 1989; Gerritsen van der Hoop et al, 1990*b*; Hovestadt et al, 1992). In a previous study, we did not establish a significant relationship between VPT and the severity of docetaxel-induced neuropathy, possibly because small fibre functions are compromised in this neuropathy (Hilkens et al, 1996). The change in VPT, in this study, can probably be accounted for by cisplatin which mainly affects large myelinated fibres.

In a phase I study on paclitaxel–cisplatin combination chemotherapy, it was suggested that the sequence of cisplatin administration before paclitaxel may be related to more profound neutropenia (Rowinsky et al, 1991). We were unable to detect differences in the severity of neurotoxicity in relation to the sequence of administration of cisplatin and docetaxel. As only 12 patients received cisplatin before docetaxel, no firm conclusions can be drawn.

In conclusion, the combination chemotherapy of docetaxel and cisplatin induces a dose-dependent sensory neuropathy. At higher dose range, neuropathy is encountered in a relatively high proportion of patients. With cumulative doses of both cisplatin and docetaxel between 200 and 600 mg $m^{-2}$, one third of the patients developed a moderate or severe neuropathy. The severity of neuropathy is higher than with the use of either cisplatin or docetaxel as a single agent at similar doses. Further study on the possible attenuating effects of neuroprotective agents such as WR-2721 (amifostine) (Mollman et al, 1988; Gandara et al, 1991; Wadler et al, 1993), glutathione (Di Re et al, 1993; Cascinu et al, 1995) and nerve growth factor (Apfel et al, 1992; Windebank et al, 1994) is warranted.

### ACKNOWLEDGEMENTS

The authors wish to thank P.J. van Assendelft for assistance in statistical analysis, Vivian van Raay for collecting parts of the data and Janet van Vliet for secretarial help.

### REFERENCES

Aamdal S, Wolff I, Kaplan S, Paridaens R, Kerger J, Schachter J, Wanders J, Franklin HR and Verweij J (1994) Docetaxel (taxotere) in advanced malignant melanoma: a phase II study of the EORTC early clinical trials group. *Eur J Cancer* **30A**: 1061–1064

Apfel SC, Arezzo JC, Lipson LA and Kessler JA (1992) Nerve growth factor prevents experimental cisplatin neuropathy. *Ann Neurol*, **31**: 76–80

Bissett D, Setanoians A, Cassidy J, Graham MA, Chadwick GA, Wilson P, Auzannet V, Lebail N, Kaye SB and Kerr DJ (1993) Phase I and Pharmacokinetic study of Taxotere (RP 56976) administered as a 24-hour infusion. *Cancer Res* **53**: 523–527

Bruntsch U, Heinrich B, Kaye SB, Mulder DE, PHM, Oosterom Van A, Paridaens R, Vermorken JB, Wanders J, Franklin H, Bayssas M and Verweij J (1994) Docetaxel (taxotere) in advanced renal cell cancer. A phase II trial of the EORTC early clinical trials group. *Eur J Cancer* **30A**: 1064–1067

Cascinu S, Cordella L, Del Ferro E, Fronzoni M and Catalano G (1995) Neuroprotective effect of reduced glutathione on cisplatin-based chemotherapy in advanced gastic cancer: a randomized double blind placebo-controlled trial. *J Clin Oncol* **13**: 26–32

© Cancer Research Campaign 1997

Chaudhry V, Rowinsky EK, Sartorius SE, Donehower RC and Cornblath DR (1994) Peripheral neuropathy from taxol and cisplatin combination chemotherapy: clinical and electrophysiological studies. *Ann Neurol* **35**: 304–311

Chevallier B, Fumoleau P, Kerbrat P, Dieras V, Roche H, Krakowski I, Azli N, Bayssas M, Lentz MA and Van Glabbeke M (1995) Docetaxel is a major cytotoxic drug for the treatment of advanced breast cancer: a phase II trial of the clinical screening cooperative group of the european organization for research and treatment of cancer. *J Clin Oncol* **13**: 314–322

DI RE F, Bohm S, Oriani S, Spatti GB, Pirovano C, Tedeschi M and Zunino F (1993) High-dose cisplatin and cyclophosphamide with glutathione in the treatment of advanced ovarian cancer. *Ann Oncol* **4**: 55–61

Elderson A, Gerritsen Van Der Hoop R, Haanstra W, Neijt JP, Gispen WH and Jennekens FGI (1989) Vibration perception and thermoperception as quantitative measurements in the monitoring of cisplatin induced neurotoxicity. *J Neurol Sci* **93**: 167–174

Extra JM, Rousseau F, Bruno R, Clavel F, Lebail N and Marty M (1993) Phase I and Pharmacokinetic study of taxotere (RP 56976, NSC 628503) given as a short infusion. *Cancer Res* **53**: 1037–1042

Fossella F, Lee JS, Murphy WK, Lippman SM, Calayag M, Pang A, Chase M, Shin DM, Glisson B, Benner B, Huber M, Perez-Soler R, Hong WK and Raber M (1994) Phase II study of docetaxel for recurrent or metastatic non-small-cell lung cancer. *J Clin Oncol* **12**: 1238–1244

Francis PA, Schneider J, Hann L, Balmaceda C, Barakat R, Phillips M and Hakes T (1994*a*). Phase II trial of docetaxel in patients with platinum-refractory advanced ovarian cancer. *J Clin Oncol* **12**: 2301–2308

Francis PA, Rigas JR, Kris MG, Pisters KMW, Orazem JP, Woollley KJ and Heelan RT (1994*b*). Phase II trial of docetaxel in patients with stage III and IV non-small-cell lung cancer. *J Clin Oncol* **12**: 1232–1237

Gandara DR, Perez EA, Wiebe V and Degregorio MW (1991) Cisplatin chemoprotection and rescue: pharmacologic modulation of toxicity. *Semin Oncol* **18**: 49–55

Gerritsen Van Der Hoop R, Van Der Burg Mel, Ten Bokkel Huinink WW, Van Houwelingen JC and Neijt JP (1990*a*). Incidence of neuropathy in 395 patients with ovarian cancer treated with or without cisplatin. *Cancer* **66**: 1697–1702

Gerritsen Van Der Hoop R, Vecht CJ, Van Der Burg Mel, Elderson A, Boogerd W, Heimans JJ, Vries EP, Van Houwelingen JC, Jennekens FGI, Gispen WH and Neijt JP (1990*b*). Prevention of cisplatin neurotoxicity with an ACTH (4–9) analogue in patients with ovarian cancer. *N Engl J Med* **322**: 89–94.

Gerven Van, JMA, Moll JWB, Bent Van Den, MJ, Bontebal M, Burg Van Der, MEL, Verweij J and Vecht Chj (1994) Paclitaxel (taxol) induces cumulative mild neurotoxicity. *Eur J Cancer* **30A**: 1074–1077

Goldberg JM and Lindblom U (1979) Standardised method of determining vibratory perception tresholds for diagnosis and screening in neurological investigation. *J Neurol Neurosurg Psychiatry* **42**: 793–803

Hilkens PHE, Planting Ast, Van Der Burg Mel, Moll Jwb, Van Putten Wlj, Vecht Chj and Van Den Bent MJ (1994) Clinical course and risk factors of neurotoxicity following cisplatin in an intensive dosing schedule. *Eur J Neurol* **1**: 45–50

Hilkens Phe, Verweij J, Stoter G, Vecht Chj, Van Putten WLJ and Van Den Bent MJ (1996) Peripheral neurotoxicity induced by docetaxel. *Neurology* **46**: 104–108

Hovestadt A, Van Der Burg Mel, Verbiest HBC, Van Putten Wlj and Vecht CJ (1992) The course of neuropathy after cessation of cisplation treatment, combined with org 2766 or placebo. *J Neurol* **239**: 143–146

Lipton RB, Apfel SC, Dutcher JP, Rosenburg R, Kaplan J, Berger A, Einzig AI, Wiernik P and Schaumburg HH (1989) Taxol produces a predominantly sensory neuropathy. *Neurology* **39**: 368–373.

Mollman JE, Glover DJ, Hogan WM and Furman RE (1988) Cisplatin neuropathy. Risk factors, prognosis, and protection by WR-2721. *Cancer* **61**: 2192–2195

New PZ, Jackson CE, Rinaldi D, Burris H and Barohn RJ (1996) Neurotoxicity of docetaxel (taxotere). *Neurology* **46**: 108–111

Pazdur R, Kudelka AP, Kavanagh JJ, Cohen PR and Raber MN (1993) The taxoids: paclitaxel (Taxol) and docetaxel (Taxotere). *Cancer Treat Rev* **19**: 351–386

Pronk LC, Schellens JHM, Planting AST, Van Den Bent MJ, Van Der Burg Mel, De Boer-Dennert M, Blanc C, Harteveld M, Bruno R, Stoter G and Verweij J (1996) A phase I and pharmacologic study of docetaxel and cisplatin in patients with advanced solid tumors. *Clin Oncol* (in press)

Roelofs RI, Hrushesky W, Rogin J and Rosenberg L (1984) Peripheral sensory neuropathy and cisplatin chemotherapy. *Neurology* **34**: 934–938

Rowinsky EK, Gilbert MR, Mcguire WP, Noe DA, Grochow LB, Forastiere AA, Ettinger DS, Lubejko BG, Sartorius SE, Cornblath Dr, Hendricks CB and Donehower RC (1991) Sequences of taxol and cisplatin: a phase I and pharmacological study. *J Clin Oncol* **9**: 1692–1703

Rowinsky EK, Chaudhry V, Forastiere AA, Sartorius SE, Ettinger DS, Grochow LB, Lubejko BG, Cornblath DR and Donehower RC (1993) Phase I and pharmacological study of paclitaxel and cisplatin with granulocyte colony-stimulating factor: neuromuscular toxicity is dose-limiting. *J Clin Oncol* **11**: 2010–2020

Smyth JF, Smith IE, Sessa C, Schoffski P, Wanders J, Franklin H and Kaye SB (1994) Activity of docetaxel (taxotere) in small cell lung cancer. *Eur J Cancer* **30A**: 1058–1060

Thompson SWE, Davis LE, Kornfeld M, Hilgers RD and Standefer JC (1984) Cisplatin neuropathy: clinical, elctrophysiologic, morphologic, and toxicologic studies. *Cancer* **54**: 1269–1275

Vecht CJ, Hovestadt A, Verbiest HBC, Van Putten Wlj, Neijt JP and Van Der Burg Mel (1991) Org 2766 in the prevention of cisplation neuropathy. In *Platinum and Other Metal Coordination Compounds in Cancer Chemotherapy* SB Howell (ed.), pp. 501–508. Plenum press: New York

Wadler S, Beitler JJ, Rubin JS, Haynes H, Mcgill F, Rozenblit A, Goldberg G, Cohen C, Speyer J and Runowicz C (1993) Pilot trial of cisplatin, radiation, and WR 2721 in carcinoma of the uterine cervix: a New York gynecologic oncology group study. *J Clin Oncol* **11**: 1511–1516

Windebank AJ, Smith AG and Russell JW (1994) The effect of nerve growth factor, ciliary neurotrophic factor, and ACTH analogs on cisplatin neurotoxicity in vitro. *Neurology* **44**: 488–494

© Cancer Research Campaign 1997